# Mesenchymal stem cell derived hematopoietic cells are permissive to HIV-1 infection

**DOI:** 10.1186/1742-4690-8-3

**Published:** 2011-01-12

**Authors:** Timo Z Nazari-Shafti, Eva Freisinger, Upal Roy, Christine T Bulot, Christiane Senst, Charles L Dupin, Abigail E Chaffin, Sudesh K Srivastava, Debasis Mondal, Eckhard U Alt, Reza Izadpanah

**Affiliations:** 1 Applied Stem Cell Laboratory, Heart and Vascular Institute, Department of Medicine, Tulane University Health Science Center; New Orleans, Louisiana, USA; 2 Department of Pharmacology, Tulane University Health Sciences Center, New Orleans, Louisiana, USA; 3 Department of Surgery, Tulane University Health Science Center; New Orleans, Louisiana, USA; 4 Department of Biostatistics, Tulane University School of Public Health and Tropical Medicine, New Orleans, LA, USA; 5 Division of Plastic and Reconstructive Surgery, Louisiana Health Sciences Center, Louisiana State University; New Orleans, Louisiana, USA

## Abstract

**Background:**

Tissue resident mesenchymal stem cells (MSCs) are multipotent, self-renewing cells known for their differentiation potential into cells of mesenchymal lineage. The ability of single cell clones isolated from adipose tissue resident MSCs (ASCs) to differentiate into cells of hematopoietic lineage has been previously demonstrated. In the present study, we investigated if the hematopoietic differentiated (HD) cells derived from ASCs could productively be infected with HIV-1.

**Results:**

HD cells were generated by differentiating clonally expanded cultures of adherent subsets of ASCs (CD90^+^, CD105^+^, CD45^-^, and CD34^-^). Transcriptome analysis revealed that HD cells acquire a number of elements that increase their susceptibility for HIV-1 infection, including HIV-1 receptor/co-receptor and other key cellular cofactors. HIV-1 infected HD cells (HD-HIV) showed elevated p24 protein and *gag *and *tat *gene expression, implying a high and productive infection. HD-HIV cells showed decreased *CD4*, but significant increase in the expression of *CCR5*, *CXCR4*, *Nef*-associated factor *HCK*, and *Vpu*-associated factor *BTRC*. HIV-1 restricting factors like APOBEC3F and TRIM5 also showed up regulation. HIV-1 infection increased apoptosis and cell cycle regulatory genes in HD cells. Although undifferentiated ASCs failed to show productive infection, HIV-1 exposure increased the expression of several hematopoietic lineage associated genes such as *c-Kit*, *MMD2*, and *IL-10*.

**Conclusions:**

Considering the presence of profuse amounts of ASCs in different tissues, these findings suggest the possible role that could be played by HD cells derived from ASCs in HIV-1 infection. The undifferentiated ASCs were non-permissive to HIV-1 infection; however, HIV-1 exposure increased the expression of some hematopoietic lineage related genes. The findings relate the importance of ASCs in HIV-1 research and facilitate the understanding of the disease process and management strategies.

## Background

Human immunodeficiency virus type 1 (HIV-1), the etiologic agent of acquired immune deficiency syndrome (AIDS), predominantly infects hematopoietic cells such as T-helper lymphocytes, monocytes and macrophages. Despite the development of highly active anti-retroviral therapy (HAART), the persistence of reservoirs of HIV-1 poses obstacles to the eradication of the disease. Although initial viral decay kinetics in plasma had indicated optimistic outcomes of HAART [1], long-term measurements have suggested that mononuclear lymphocytes harbor the virus for prolonged periods of time [2].

Infection of lymphoid and myeloid lineages is mediated by recognition of the T-cell receptor CD4 or by the chemokine co-receptors CXCR4 and CCR5. CXCR4 appears to be the most important for HIV-1 entry into T-lymphocytes (T-tropic), whereas CCR5 is known for viral entry into cells such as monocytes and macrophages (M-tropic) [3]. These receptors promote viral attachment and fusion to cellular membranes, thus facilitating entry into hematopoietic cells [4]. Although the peripheral blood-derived hematopoietic progenitor cells (HPCs) can express the HIV-1 co-receptors [5], susceptibility to either T-tropic or M-tropic strains of HIV-1 seem to correlate only with lineage commitment of HPCs [6]. Even though an early loss of circulating CD34^+ ^HPCs and impaired clonogenic potential and apoptosis of these progenitor cells have been documented in HIV-1 infected individuals [7,8], the evidence of productive infection of HPCs remains controversial [9,10].

The mesenchymal stem cells (MSCs) are endowed with multi lineage differentiation potentials and self-renewal properties, which qualify them as potential sources for cell transplantation and gene therapy. MSCs from several origins, including bone marrow and adipose tissue, have been well described. Adipose tissue derived MSCs (ASCs), like bone marrow derived MSCs, have the capacity to differentiate along multiple lineages at clonal levels. They can differentiate into neurons, cardiomyocytes, chondrocytes, osteocytes, and adipocytes [11-16]. However, it is not known whether lineage specific differentiation of MSCs would enable them to be infected by HIV-1 and whether they may act as long-term viral reservoirs within systemic sites.

The HIV-1 infection of bone marrow mesenchymal progenitors and of mesenchyme-derived cells (e.g., fibroblasts and endothelial cells) present in various peripheral organs has been shown to occur via both M-tropic and T-tropic strains of HIV-1 [17-19]; however, integrated provirus is rarely found in these cells and a productive infection has not been documented. However, *in vitro *infection of stromal cells grown in long-term bone marrow cultures (LTBMC) with HIV-1 has been reported [20-22]. Our previous studies had shown that a T-tropic strain of HIV-1 can infect bone marrow MSC cultures and decrease their colony forming ability and adipogenic potential [23]. Further, it has also been shown that multipotent human progenitor cells isolated from fetal brains are permissive towards HIV-1 infection [24]. However, it has not been well established as to how these mesenchyme derived cells become susceptible to HIV-1 and whether their HIV-1 production rates are comparable to that observed in HIV-1 infected lymphoid or myeloid cells. Importantly, despite the possible presence of ASCs in systemic organs, there is no evidence about the ability of HIV-1 to infect either undifferentiated ASCs or their differentiated counterparts.

Recent work from our laboratory has demonstrated that under specific *in vitro *stimulations even the CD34^- ^ASC clones (CD90^+^, CD105^+^, CD45^- ^and CD34^-^) could undergo hematopoietic differentiation (HD) and display macrophage-like characteristics [25]. Macrophages are known to play a crucial role in both HIV-1 infectivity and pathogenesis. Although they can generate high levels of viral progeny, they are resistant to HIV-1 induced cytopathic effects and harbor the virus for a long time [26-28]. Hence, our efforts were focused on studying the susceptibility of the ASCs and the HD-differentiated ASCs for HIV-1 infection and their subsequent abilities to support viral replication. Initially, the differentiated cells were analyzed for receptors, ligand binding, and cofactors, which are directly involved in HIV-1 infection, followed by analysis of changes in gene expression that occurs following HIV-1 infection. Both HIV-1 susceptibility markers and productive replication in HIV-1 exposed HD cells were compared with those observed in a HIV-1 infected T-cell line, and the findings are reported in the present study.

## Results

### Up-regulation of HIV-1 susceptibility genes in HD cells

**Figure 1:**
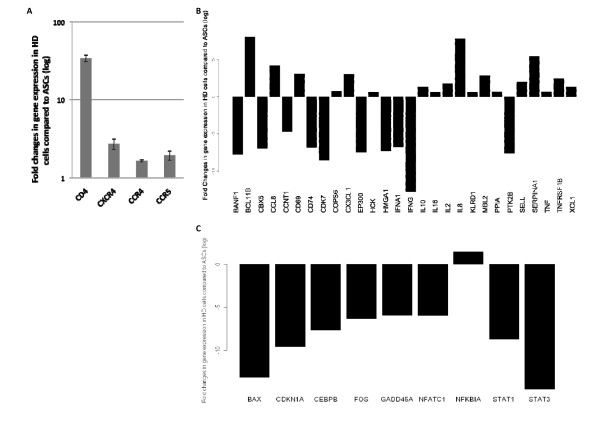
**Gene expression analysis of HD cells following hematopoietic differentiation**. Compared to ASCs, the expression level of HIV receptor genes (*CD4, CXCR4, CCR4*, and *CCR5*) were up regulated in HD cells as result of differentiation **(A)**. Expression of several genes involved in innate and adaptive immune reactions **(B)**, and cell cycle regulators **(C) **were altered in HD cells. A fold change was applied to select genes (P < 0.05). All values are normalized to ASCs (X axis).

HD cells were prepared by differentiating expanded cultures of ASC clones, phenotypically identified as CD90^+^, CD105^+^, CD44^+^, CD4^-^, CD68^-^, CD34^-^, CD45^-^, and CD11b^- ^cells as described previously [25]. For the initial assessment of HD cells, we performed a transcriptomic analysis after 8 days of differentiation. The HD cells expressed a number of HIV-1 receptors such *CD4 *(33.9 ± 3.4 fold), *CXCR4 *(2.7 ± 0.42 fold), *CCR4 *(1.64 ± 0.05 fold), and *CCR5 *(1.93 ± 0.26 fold) compared to undifferentiated ASCs (Figure 1A). HD cells also expressed a series of genes involved in innate and adaptive immune reactions and key cellular cofactors for HIV-1 infection such as *IL-8*, *SERPINA1*, *CCL8*, *CD69 *and interleukins 2, 10, and 16 (Figure 1B). The expression of lymphoid associated gene *BCL11B *was markedly up regulated. Further, the expression of a number of cell cycle regulators, such as *BAX*, *CDKN1A*, *FOS*, *GADD45A*, *NFATC1*, *CEBPB*, *STAT1*, and *STAT3*, decreased while the expression of *NFκB1A *slightly increased as a result of differentiation (Figure 1C).

### A highly productive HIV-1 infection is evident in virus exposed HD cells

**Figure 2:**
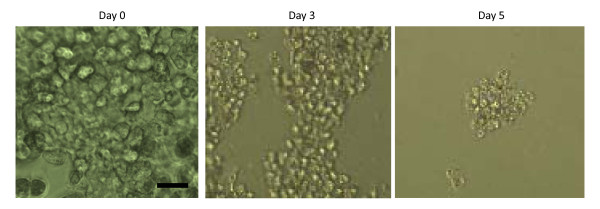
**Morphological changes in differentiated HD cells following exposure to HIV-1**. Day-0, HD cells which are on day 8^th ^of differentiation and before viral infection. Day-3, shows the morphological changes of HD cells following 72 hours of viral infection. Day-5, morphology of HD cells infected with HIV-1 virus following 120 hours of infection. Images were taken with Nikon Eclipse 2000. Scale bar is 100 μm.

**Figure 3:**
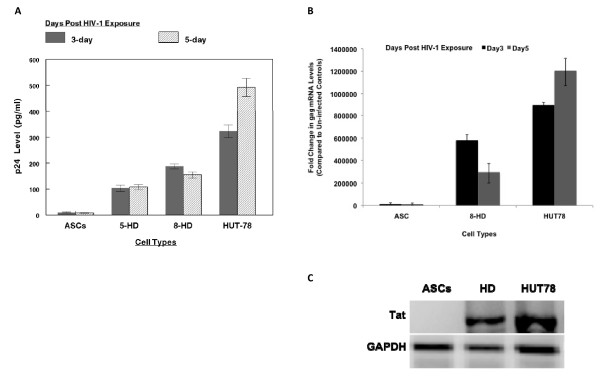
**Expression of HIV-1 p24 protein in HD-HIV and ASC-HIV cells.** p24 antigen level was monitored following post exposure to HIV-1 for 24 hr. HIV-1 was exposed to undifferentiated, 5-HD and 8-HD cells and p24 level was measured after 1, 3 and 5 days following removal of virus **(A)**. Data represent the compilation of three separate experiments carried out in triplicates (P < 0.0001). **(B)** HIV-1 *gag *expression in HD-HIV and HUT78-HIV cells and compared to HIV-1 exposed ASCs (P < 0.05). **(C)** mRNA was extracted from ASC-HIV, HD-HIV, and HUT78-HIV cells and RT-PCR was performed for *Tat *expression.

Since cells of the hematopoietic system are among the main targets of the HIV-1 virus, we investigated the effect of viral exposure on HD cells. Clonally expanded cells were allowed to differentiate into HD cells for 5 (5-HD) or 8 (8-HD) days in differentiation media. For analyzing the infectivity of HD cells, we exposed them to very low levels of HIV-1 virus (10^3^-10^4 ^TU/10^5 ^cells or 0.1 MOI) for 24 hours. Unbound viral particles were removed and cultures maintained for an additional 5 days. Following infection of HD cells, noticeable morphological changes beginning from day 3 post-infection were observed. These morphological changes heralded a loss of significant numbers of cells by day 5 post-infection, indicating the dominance of viral infection on HD cells (Figure 2). Subsequently using ELISA for HIV-1 p24, we assayed the levels of HIV-1 p24 released in the supernatant of HD-HIV, and HIV-1 exposed undifferentiated ASCs (ASCs-HIV) cultures which served as controls. The concentration of p24 in culture supernatant is depicted in Figure 3A.

Both 5-HD and 8-HD cultures showed consecutively increasing levels of p24 on days 3 to 5. On day 5 of infection, p24 levels in 5-HD and 8-HD cultures remained unchanged; the p24 levels in ASCs-HIV were negligible, indicating no evidence of viral replication.

To quantify HIV-1 cDNA and proviral DNA, the mRNA level of "*gag*" and "*Tat*" were assayed. Figure 3B shows the increased expression of *gag *in HD-HIV cells 3 days post infection. This level decreased significantly by day 5 after infection. The *gag *expression in HD-HIV cells was comparable to the HIV-1 infected "HUT-78" (HUT78-HIV), a T-lymphoblastoid cell line that served as a positive control in these experiments. The RT-PCR experiments showed enhanced expression of *Tat *in HD-HIV and HUT78-HIV cells (Figure 3C). The expression of *gag *and *Tat *were not detected in ASCs-HIV.

### HIV-1 infection significantly alters the gene expression profile in HD cells

**Figure 4:**
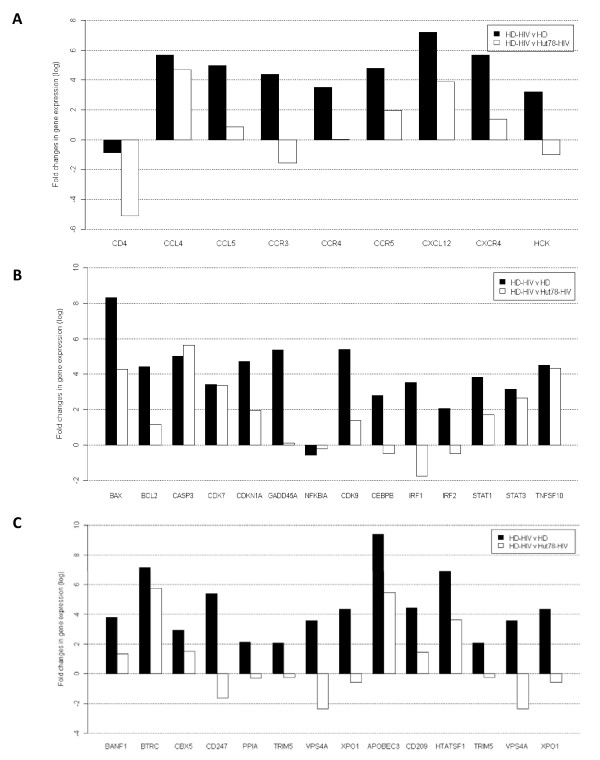
**Comparison of the gene expression profiles of HIV-1 infected HD and HUT78 cells**. Genes that were found to be differentially expressed in HD-HIV vs. HD cells in one set and HD-HIV vs. HUT78-HIV cells in another set were then grouped according to functional categories including genes encoding for HIV-1 receptors and ligands (A), cell cycle and apopotosis (B), and cellular factors involved in HIV-1 infection (C). A fold change was applied to select genes (P < 0.05). All values are normalized to either ASCs or HD cells (X axis).

The expression of selected genes mainly involved in HIV-1 infection and immune response was analyzed as described in the methods. The results obtained for each group, normalized to the mean value of the house keeping gene, were compared by scatter plot analysis using PCR-array data analysis software (SABiosciences). To study the effect of viral exposure on HD cells, we compared the expression of selected genes in HD-HIV cells versus un-infected HD cells. The gene profile of HD-HIV cells was then compared to HUT78-HIV cells. The analysis showed that HIV-1 infection altered gene expression within HD cells in a similar fashion to that seen in HUT78-HIV cells. Several genes were perturbed in response to viral exposure, and these included genes coding for HIV-1 receptors and ligands (*CCL4, CCL5, CCR5, CXCL12, CXCR4, CXCL12*). The viral exposure showed its maximum effect on the HD-HIV cells, when compared to HUT78-HIV cells (Figure 4A).

HIV-1 infection also profoundly altered expression of the cell cycle and apoptosis regulatory genes including *BAX, BCL2, CDKN1A, GADD45A, CDk9, IRF1, CEBPB*, and *IRF2*. The changes in the expression levels of these genes were more pronounced in HD-HIV cells when compared to HUT78-HIV. However, there were smaller differences in the expression levels of *Caspase 3, NFκBIA, TNSF10, STAT1*, and *STAT3 *(Figure 4B).

HIV-1 infection caused significant changes in the expression of cellular factors involved in HIV-1 infection such as *BANF1, CD247, TRIM5, VPS4A, XPO1, CD209 *and to a lesser extent, changed the expression levels of a β-transducin repeat containing (*BTRC*), *CBX5*, and *HTATSF1*. HD cells showed enhanced expression of genes coding for factors known to restrict HIV-1 replication such as *CD209, APOBEC3F*, Tat specific factor 1 (*TAT-SF1*), and tripartite motif-containing 5 (*TRIM5*) (Figure 4C).

**Figure 5:**
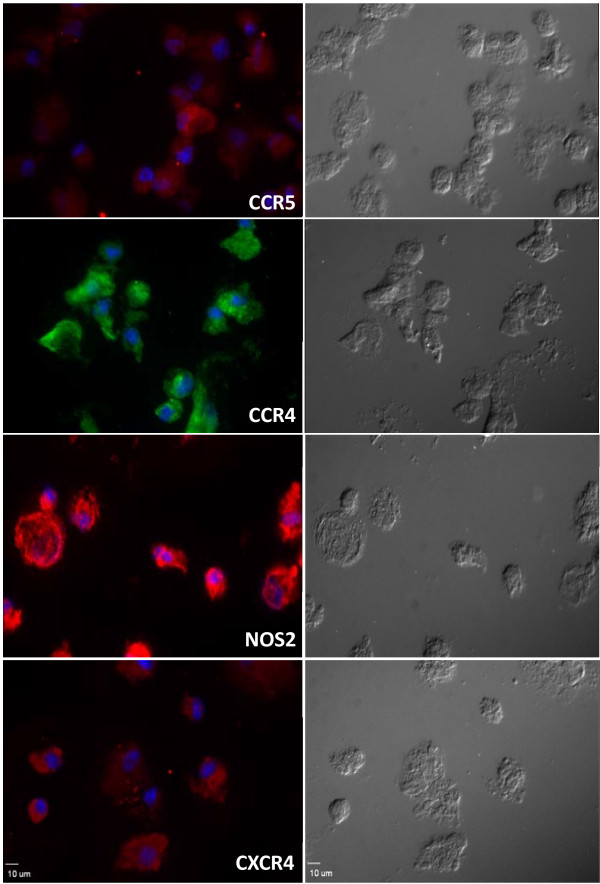
**Expression of hematopoietic markers in HD cells following HIV infection**. Immunohistochemistry of HD-HIV cells, fluorescent images indicate the expression of CCR5, CCR4, NOS2, and CXCR4. Right panel shows the DIC images of identical fields. Images were obtained with Leica TCS SP-2 confocal microscope. Scale bar 10 μm.

Immunocytochemistry was employed to analyze the expression of CCR4, CCR5, NOS2 and CXCR4 proteins in HD-HIV cells. As shown in Figure 5, these markers could be readily detected in approximately all cells. However, the expression of CD4 was in undetectable levels by immunohistochemistry

### Productive infection is not seen in undifferentiated ASCs

**Figure 6:**
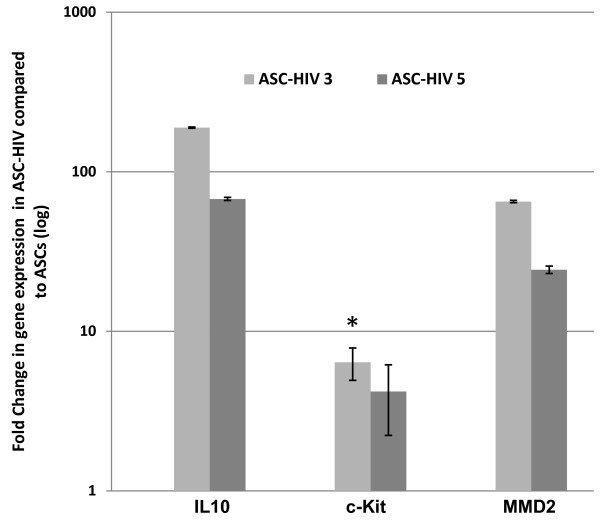
**Effects of HIV-1 exposure on gene expression of ASCs**. The gene expression levels were calculated based on the dCT-values which have been standardized with the GADPH levels. The ASCs (n = 4 donors) were harvested following 3 and 5 days after exposure to HIV-1. *IL10*, *c-Kit*, and *MMD2 *expression increased significantly by 3 days post HIV-1 exposure (P < 0.05, * represents P < 0.01). The ASCs showed no morphological changes during the course of experiment.

Undifferentiated ASCs exposed to HIV-1 resulted in no significant productive infection up to 5 days. In addition, viral exposure did not cause noticeable effects on the viability of ASCs. Exposure to low MOI (0.1) of the virus did not show any significant effect on the expression of *CD4, CD14, CD68, MSR1, TNFα *and *MRC1 *in ASCs. However, exposure to HIV-1 resulted in a provisional up-regulation of *c-Kit *(6.4 ± 1.4 fold, p ≤ 0.05), *IL10 *(188.9 ± 1.6 fold, p ≤ 0.01) and *MMD2 *(65 ± 1.1 fold, p ≤ 0.01) by day 3 post-exposure. By day 5, the expression of these genes decreased, however, the levels were still higher than in the un-exposed control ASCs (*IL10 *= 67.5 ± 1.5, p ≤ 0.01; *MMD2 *= 24.3 ± 1.3, p ≤ 0.01; and *c-Kit *= 4.1 ± 1.9, p ≥ 0.05). The decline in the expression of *MMD2 *on day 5 as compared to day 3 was significant (p ≤ 0.01) (Figure 6). Our observations indicate that the HIV-1 exposed ASCs showed significantly lower adipogenic, osteogenic potential. However, HIV exposure seems to expedite the generation of HD cells to less than 5 days (from the normal 8 days), when placed in hematopoietic differentiation media. The generated HD cells from HIV-1 exposed ASCs did not exhibit any evidence of productive infection.

The possible integration of HIV-1 in an exposed ASC genome was examined by repetitive-sampling *Alu-gag *PCR technique described earlier [29]. Briefly, on a nested based PCR technique, the regions of varying length between genomic *Alu *repeats and the HIV *gag *were amplified from the DNA of exposed ASCs to 0.1 MOI of HIV-1 for 24 h. Following this, the second PCR was performed on specific regions of the HIV-1 genome. No evidence of HIV-1 integration was observed in exposed ASCs.

## Discussion

In postnatal and adult life, macrophages differentiate from progenitor cells through various pathways. Macrophages are known to be one of the most important targets for HIV-1 infection and play a crucial role in both viral latency and recrudescence. The CD34^+ ^hematopoietic progenitor cells are commonly known to generate macrophages. Furthermore, the ability of embryonic stem cells to generate HIV-1 susceptible macrophages has been reported [30]. For the first time we showed that a clonally expanded CD90^+^, CD105^+^, CD44^+^, CD4^-^, CD34^-^, CD45^-^, CD11b^-^, CD68^- ^subset of ASCs could also generate cells with macrophage attributes [25]. In the present study, we show that the generated HD cells support productive HIV-1 infection. We show the expression of several HIV-1 susceptibility genes as well as several immune response genes in HD cells. A number of such newly expressed genes may possibly be involved in increasing the susceptibility of HD cells to HIV-1 infection.

Previously we showed that early in differentiation, HD cells develop CD4, a T-lymphocyte marker [25]. Since we utilized the HTLV-III_B _strain which is a T-tropic virus, the infectivity in HD cells as compared to the newly infected HUT78 cells can be explained by utilizing CD4 as one of the most important HIV-1 receptors. Furthermore, as a result of differentiation, expression of other common cellular ligands essential for HIV-1 infection, such as *CXCR4, CCR4*, and *CCR5*, distinctly increased. In addition, the expression of markers associated with activated immune cells, such as the serine protease inhibitor *SERPIN-A1*; the cell surface markers such as *CCL8, CD69*; as well as the expression of interleukins such as *IL-2, IL-8, IL-10*, and *IL-16*, were markedly increased in the HD cells. These observations clearly indicated that these mesenchymal origin cells acquired the attributes of hematopoietic cells.

Our current findings clearly demonstrate a profound increase in the susceptibility of HD cells to HIV-1 infection. Interestingly, higher levels of p24 expression were observed in 8-HD compared to 5-HD cells. This is suggestive that 8-HD cells develop even more cellular receptors for viral entry and are prone for replication. The negligible amount of p24 in ASCs-HIV might be associated with a residual amount of virus floating in the media. Compared to HUT78 cells, the HD cells supported a highly productive HIV-1 infection as evident from the significantly higher levels of *gag *and *Tat *expression in both cell types post HIV-1 exposure. The *gag *expression level decreased in HD-HIV on day-5 post infection which was due to considerable cytotoxicity associated with the HIV infection (Figure 3B).

In order to demonstrate the infectivity of HD cells, we exposed them to very low levels of the virus (0.1 MOI). Indeed, similar to the high level of infectivity observed in the HUT78 cells, the HD cells showed increasing levels of p24 in the supernatants on both day-3 and day-5 post infection, suggesting a possible and crucial role *in vivo *in providing infectable cells.

Interestingly, HIV-1 exposed cells showed a significant decrease in expression of *CD4*. Significant increases in the expression of HIV-1 co-receptors, both CCR5, and CXCR4 were observed in the HD-HIV cells in both gene and protein levels. Although we have not carried out studies using an M-tropic virus, our findings point towards virus infection that enables HD cells to be susceptible to both of R5 and X4 viruses. Previous reports determined that the HIV-1 Tat protein alters co-receptor expression in lymphoid and myeloid cells [31,32]. Studies from our laboratory on the HPC cell line, K562 had also indicated that Tat can differentially regulate both CXCR4 and CCR5 expression in erythroid or megakaryocytic cells [33]. Although we have not measured Tat expression in the HD-HIV cells, the level of productive infection clearly suggests high levels of Tat protein which may be directly involved in changes in HIV-1 receptor expression observed in these cells.

As compared to the newly infected HUT78-HIV cells, the HD-HIV cells showed significantly higher level of expression of several chemokines such as *CXCL12*, *CCL4 *and *CCL5*. CCL4 (MIP-1β) is a major HIV-suppressive factor produced by CD8^+ ^T cells [34]. It has also been documented that MIP-1α and RANTES, as ligands for CCR5, may suppress HIV-1 infection as well [35]. An increase in CXCL12 (SDF-1α) in lymphocytes has also been associated with decreased infectivity of HPCs via the X4-tropic strains of HIV-1 [32]. In HD-HIV cells, the increased expression of several chemokines may thus suggest that the cells are combating to inhibit virus infection by producing these ligands which compete for HIV-1 binding to cells. In addition, this may also suggest that by secreting these chemokines the HD-HIV cells may enhance the recruitment of virus infectable cells to the microenvironments *in vivo*. Although we have not measured the levels of these chemokines produced from the HD-HIV cells, an increase in their gene expressions of almost over 10 fold indicates their protein levels may also be augmented and may therefore play a crucial role in the development of HIV-1 reservoirs.

Although the levels of productive infection (both p24 protein, *gag *and *Tat *mRNA levels) were almost similar in the HD-HIV and HUT78-HIV cells, there were several other salient differences in the gene expression profiles following HIV-1 infection. In addition to the differences in chemokine and their receptor expressions (Figure 4A), significant differences were seen in several apoptotic markers (Figure 4B) and in several lineage specific transcription factors (Figure 4C). In the HD cells, HIV-1 infection altered the expression of genes associated with apoptosis such as *BAX, BCL2, CASP3 *and *GADD45α*. HD cells also exhibited elevated levels of *BCL11B*, a transcription factor expressed in T-cells [36]. Interestingly, BCL11B has been found to repress HIV-1 transcription from the 5' long terminal repeat [37]. Genes associated with cell cycle regulation, such as *CDKN1A, CDK7 *and most importantly *CDK9*, were also up regulated in the HD-HIV cells, as compared to uninfected HD as well as HUT78-HIV cells. Since, CDK9 plays a crucial role in HIV-1 Tat protein mediated transactivation, a possible role of increased Tat function in the productive infection may also be considered likely. Indeed, the expression of several other transcription factors that are also known to regulate HIV-1 promoter activity, e.g. *CEBP-β *and both *STAT1 *and *STAT3 *genes, were also up regulated in HD-HIV cells, as compared to uninfected HD as well as HUT78-HIV cells. Interestingly, the mRNA expression of *NFκB1A*, the p65 subunit of the transcription factor NFκB which also regulates HIV-1 gene expression in stimulated lymphocytes, was not decisively altered in these cells. However, several cell surface receptors that regulate intracellular NFκB activity, such as TNFSF10 and both IRF1 and IRF2 were higher.

The up-regulation of *Bax*, which results in a loss of mitochondrial membrane polarization and release of pro-apoptotic factors culminating in caspase activation and apoptosis, has been documented [38]. Andersen and coworkers have reported that the expression of *GADD45A *was increased following stressful growth arrest conditions as a result of HIV-1 infection [39]. HIV-1 has also been shown to regulate the expression of *CDK9 *[40]. It has been shown that *STAT3 *promotes the initiation of transcription and regulates chromatin remodeling and transcription elongation through its interaction with *CDK9 *[41].

We also found that the expression of several genes such as *BANF1, BTRC, CD209, APOBEC3F*, and *TAT-SF1 *increased in HD-HIV cells. *BANF1 *is known for its ability to protect retroviruses from intra-molecular integration and there by promoting intermolecular integration into the host cell genome [42]. BTRC interacts with HIV-1 viral protein U (Vpu) and connects CD4 to the proteolytic machinery [43]. *CD209 *expression has been reported in association with HIV-1 infection [44]. APOBEC3F potently restrict HIV-1 replication, and is neutralized by the viral protein Vif [45]. Increased *TAT-SF1 *expression in HD-HIV cells was significant and since it has been shown that *TAT-SF1 *is required for maintaining the ratios of different classes of HIV-1 transcripts [46]. These findings suggest that pathways that facilitate productive infection in T-cells may also be induced in the HD-HIV cells, as is clearly evident from the levels of p24 and *gag *and *Tat *expression in both cell types.

In the present studies for the first time, the infective property of HIV-1 on cells derived from ASCs is reported. It has been reported that HIV-1 stimulates the secretion of the adipocyte-derived hormone adiponectin, however no evidence of infectivity of the virus on adipocytes were shown [47]. Our studies show that ASCs respond to HIV-1 exposure by increasing expression of *IL-10*, *c-Kit*, and *MMD2*. Although these effects do not ultimately result in productive infection, data revealed that HIV-1 exposure increases the hematopoietic lineage commitment of ASCs. The enhanced hematopoietic capacity of HIV-1 exposed ASCs was concurrent with decline in their adipogenic and osteogenic potential. Recently, it has been reported that chronic exposure of CD4^+^, CXCR4^+^, and CCR5^+ ^mesenchymal stem cells with high viral load sera enhanced the adipogenesis [48]. While the treatment of cells with low viral load did not alter differentiation potential of those cells, the ASC clones used in this study were negative for *CD4, CXCR4*, and *CCR5*. In addition, our data suggest no HIV-1 integration into the ASC genome. These results of the present study shed light on the effect of HIV-1 on tissue resident stem cells paving way for additional studies to explore the mechanistic insights for understanding and management of the disease process.

## Conclusion

Based on the observations reported, it is now feasible to study the effect of anti-HIV treatments on ASC derived HD cells. The presence of phenomenal numbers of ASCs in adipose tissue, and these novel findings which indicate that HIV-1 exposure may facilitate their macrophage type commitment, demonstrates that these cells may have importance in generating systemic viral reservoirs. Further, the utility of ASC as well as the ASC derived HD cells as a possible tool for future gene therapy against HIV-1 seems to be promising and merits additional investigation.

## Methods

### Cell culture and Hematopoietic Differentiation

All human tissue sample collection protocols were reviewed and approved by Institutional Review Board (IRB) of Tulane University. Human ASCs were isolated from adipose tissue of healthy donors (n = 14) based on the methods described earlier [49]. ASCs clones were isolated and expanded in α-MEM (CellGro, Manassas, VA) based media, supplemented with 20% fetal bovine serum (Atlanta Biologicals, Lawrenceville, GA), 1% L-Glutamine (CellGro, Manassas, VA) and 1% penicillin/streptomycin (CellGro, Manassas, VA) at 37°C in 5% CO_2_. Then clones were cultured in differentiation media according to a previously described method [25]. Briefly, clonally expanded ASCs were plated at a density of 5000 cells/cm^2 ^on either cell culture dishes or chamber slides (Nalgene, Nunc, Rochester, NY). The differentiation media consisted of α-MEM, 10% FBS, 0.1 μl/ml 1-monothioglycerol (MTG) (Sigma-Aldrich Inc. St. Louis, MO), supplemented with 100 U/ml IL-1ß, 500 U/ml IL-3, and 20 U/ml M-CSF (Prospec Bio, Rehovot, Isreal) as stimulating substances. 30% of the primary volume was augmented with fresh media every 2 days for 12 days. Cultures of ASC clones in growth media containing 10% FBS served as control undifferentiated cells.

### HIV-1 Infection

The uninfected T4-lymphocyte line HUT78, and HUT78 cells persistently infected with HTLV-III_B _strain of HIV-1 (from the AIDS Research & Reference Reagent Program, Bethesda, MD) were cultured in RPMI medium, supplemented with 10% FBS and antibiotics (penicillin & streptomycin). Cell-free viral stocks were obtained from the supernatants of HTLV-III_B _infected cell line grown to 50-60% confluency. The viral titers were determined by measuring HIV-1 p24 levels using an ELISA kit as per the manufacturer's protocol (ZeptoMetrix, Buffalo, NY). For ASC infection studies, cells were cultured in differentiation media for either 5 (5-HD) or 8 (8-HD) days, and subsequently exposed to cell free virus for 24 hr. For HUT-78 cell infection, uninfected cells growing in the logarithmic phase were exposed to cell free virus for 24 hrs. All HIV-1 exposure studies were performed using a viral stock of ~100 pg/ml of p24 [10^3^-10^4 ^transducing units (TU)/ml]. Each viral stock was freshly prepared before exposure of ASCs or uninfected HUT78 cells. For controls, un-differentiated ASCs and HUT78 cells were exposed to the same number of viral particles. Following 24 hrs of virus exposure, cells were washed several times using fresh media to remove the unattached viral particles and cultured for 3 or 5 days post exposure. Prior to viral infection, ASCs were cultured in differentiation media for either 5 or 8 days, and subsequently exposed to HIV-1 in cell free viral media. Viral p24 levels were analyzed at each time point to monitor the viral replication in un-differentiated, HD, and HUT78 cells. A graphical representation of p24 level (pg/ml) vs time point (days) was carried out. Values with p < 0.0001 were considered significant.

### Alu-gag PCR

The genomic DNA from exposed HIV-1 ASCs was subjected to two step *Alu-gag *PCR technique described by Liszewski *et al*. [29]. In the first step the *Alu-gag *regions were amplified using following primers: 1. *Alu *(Forward): 5' GCC TCC CAA AGT GCT GGG ATT ACA G-3'; HIV *gag *(Reverse): nucleotides (nt) 1505-1486 5' GTT CCT GCT ATG TCA CTT CC-3'. On the second step, RU5 region in *gag *was detected using the following primers: RU5 (R Forward): nt 518-539 5'-TTA AGC CTC AAT AAA GCT TGC C-3'; RU5 (U5 Reverse): nt 647-628 5'-GTT CGG GCG CCA CTG CTA GA-3'; 5. RU5wildtype Probe: nt 584-559 5'-CCA GAG TCA CAC AAC AGA CGG GCA CA-3'; RU5degenerate1 Probe: nt 584-559 5'-CCA GAG TCA CAT AAC AGA CGG GCA CA-3'; and RU5degenerate2 Probe: nt 584-559 5'-CCA GAG TCA CAC AAC AGA TGG GCA CA-3'. The PCR products were analyzed by agarose gel electrophoresis.

### RT^2 ^qPCR gene expression analysis

Using total cellular RNA the gene expression was carried out on PCR array kits (SABiosciences, Frederick, MD) which profiles the expression of 84 genes involved in susceptibility to HIV-1, infection and related immune response. The cellular RNAs from un-differentiated ASCs, HD, and HIV-1 infected HD cells (HD-HIV) (n = 3 donors) with HIV-1 infected HUT78 (HUT78-HIV) (serving as positive controls) were used. Data were analyzed using software provided by SABiosciences http://www.sabiosciences.com. Differential gene expression was evaluated for statistical significance (p < 0.05). A cut off of 2 for fold change for up-regulated and 0.5 for down regulated genes was applied, so as to only consider genes whose expression was perturbed in magnitude as well as in a significant manner.

### RT-PCR and Real-Time RT-PCR

Real-Time RT-PCR was performed using SYBR Green Master mix (Invitrogen, Carlsbad, CA) in a 2-step protocol (50 cycles of 10 sec at 90°C and 45 sec at Tm). The following primers were used to assess the gene expressions. *CD4*: 5'-GTA GTA GCC CCT CAG TGC AA-3', 5'-AAA GCT AGC ACC ACG ATG TC-3'; *CD14*: 5'-ACA GGA CTT GCA CTT TCC AG-3', 5'-TCC AGG ATT GTC AGA CAG GT-3'; *CD68*: 5'-CAA CTG CCA CTC ACA GTC CT-3', 5'-CAA TGG TCT CCT TGG AGG TT-3'; *IL10*: 5'-AAG CCT GAC CAC GCT TTC TA-3', 5'-ATG AAG TGG TTG GGG AAT GA-3'; *ITGAM*: 5'-ACG GAT GGA GAA AAG TTT GG-3', 5'-CAA AGA TCT TCT CCC GAA GC-3'; *c-KIT*: 5'-CCG TGG TAG ACC ATT CTG TG-3', 5'-GTG CCC ACT ATC CTG GAG TT-3'; *MMD2*: 5'-GCA GAC CAA GGT GTC CAA AT-3', 5'-CTG GCT GTC ACC AGA AGT CA-3'; *MRC1*: 5'-GGC GGT GAC CTC ACA AGT AT-3', 5'-ACG AAG CCA TTT GGT AAA CG-3'; *MSR1*: 5'-TCC TCG TGT TTG CAG TTC TC-3', 5'-CAT GTT GCT CAT GTG TTC CA-3'; *TNF*: 5'-TCC TTC AGA CAC CCT CAA CC-3', 5'-AGG CCC CAG TTT GAA TTC TT-3'; *gag*: 5'-ATA ATC CAC CTA TCC CAG TAG GAG AAA T-3', 5'-TTT GGT CCT TGT CTT ATG TCC AGA ATG C-3'; *Tat: *5'-GGA ATT CAC CAT GGA GCC AGT AGA TCC T-3', 5'-CGG GAT CCC TAT TCC TTC GGG CCT GT-3'; *GAPDH *5'-CGA GAT CCC TCCA AAA TCA A-3' and 5'-GGT GCT AAG CAG TTG GTG GT-3'. The data were generated using an iCycler MyiQ (Biorad) and analyzed using the iQ5 V2.0 (Bio-Rad). The RT-PCR products were analyzed using agarose gel electrophoresis (1% agarose gel) and stained in 10 μg/ml ethidium bromid (Sigma) for visualization. For real-time RT-PCR, the equation 2^-ΔΔCT ^was used for calculating fold changes. A threshold cycle of 35 was chosen as the cut-off for non-detectable genes, thus genes with CT values above 35 were considered not expressed.

### Immunocytochemistry

HD cells were prepared and infected with HIV, the fixed, permeabilized, and incubated with human specific primary antibodies for CCR4, CCR5, CXCR4, and NOS2 at a final concentration of 0.02-0.04 mg/ml, then incubated with 0.002 mg/ml of the matching secondary antibody. The signal was detected with a Leica TCS SP-2 confocal microscope equipped with Argon (457-477 nm; 488 nm, 514 nm) and HeNe lasers (543 nm; 633 nm) at a magnification of HCX PL APO 63×/1.4 at 21°C. Data were processed with Leica confocal software.

### Osteogenic and Adipogenic Differentiation

Adipogenic differentiation was determined in cultures of ASCs following HIV exposure using previously described methods [16]. Adipogenic potentials were evaluated by oil red O staining. Osteogenic differentiation was induced as previously described [50]. Differentiated cells were either fixed and stained with Alizarin Red (Diagnostic BioSystems) or quantified for alkaline phosphatase activity (ALP) using the SensoLyte™ pNPP Alkaline Phosphatase Assay Kit (AnaSpec, San Jose, CA). All analyzes were carried out in triplicates.

### Statistical analysis

All data relating to this study were summarized using descriptive statistics such as mean, standard deviation and standard error. The analysis of variance method was used to compare the mean differences. Where meaningful, the results were presented graphically. The study hypotheses were tested at 5% level of significance throughout the analysis. Estimates of means and their 95% confidence intervals were calculated. R-computing software was used to plot the graphs.

## Abbreviations

MSCs: mesenchymal stem cells; ASCs: adipose tissue derived mesenchymal stem cells; HD: hematopoietic differentiated cells; AIDS: acquired immune deficiency syndrome; HAART: highly active anti-retroviral therapy; HPCs: hematopoietic progenitor cells; LTBMC: long-term bone marrow cultures; HD-5: differentiate into HD cells for 5 days; HD-8: differentiate into HD cells for 8 days; MOI: multiplicity of infection; HD-HIV: infected HD cells with HIV-1; HUT78-HIV: infected HUT78 cells with HIV-1; ASCs-HIV: exposed ASCs to HIV-1; TRIM5: tripartite motif-containing 5; TAT-SF1: Tat specific factor 1; BTRC: b-transducin repeat containing.

## Competing interests

The authors declare that they have no competing interests.

## Authors' contributions

EF was responsible to cell cloning and cultures. EF, TN, and CS conducted differentiations. UR and CB were responsible for HIV-1 infection studies. AC and CD were responsible for tissue collections. RI, DM, and EA were responsible for experimental design. RI and EA were responsible for the overall experimental design and implementation of the project and contributed equally to this work.

## References

[B1] Perelson AS Essunger P Ho DD Dynamics of HIV-1 and CD4+ lymphocytes in vivo AIDS 1997 11 Suppl A S17 24 9451962

[B2] Ogg GS Jin X Bonhoeffer S Moss P Nowak MA Monard S Segal JP Cao Y Rowland-Jones SL Hurley A Markowitz M Ho DD McMichael AJ Nixon DF Decay kinetics of human immunodeficiency virus-specific effector cytotoxic T lymphocytes after combination antiretroviral therapy J Virol 1999 73 797 800 10.1128/jvi.73.1.797-800.1999 PMC103892 9847391

[B3] Moore JP Kitchen SG Pugach P Zack JA The CCR5 and CXCR4 coreceptors--central to understanding the transmission and pathogenesis of human immunodeficiency virus type 1 infection AIDS Res Hum Retroviruses 2004 20 111 126 10.1089/088922204322749567 15000703

[B4] Zaitseva M Peden K Golding H HIV coreceptors: role of structure, posttranslational modifications, and internalization in viral-cell fusion and as targets for entry inhibitors Biochim Biophys Acta 2003 1614 51 61 10.1016/s0005-2736(03)00162-7 12873765

[B5] Ruiz ME Cicala C Arthos J Kinter A Catanzaro AT Adelsberger J Holmes KL Cohen OJ Fauci AS Peripheral blood-derived CD34+ progenitor cells: CXC chemokine receptor 4 and CC chemokine receptor 5 expression and infection by HIV J Immunol 1998 161 4169 4176 9780190

[B6] Chelucci C Casella I Federico M Testa U Macioce G Pelosi E Guerriero R Mariani G Giampaolo A Hassan HJ Peschle C Lineage-specific expression of human immunodeficiency virus (HIV) receptor/coreceptors in differentiating hematopoietic precursors: correlation with susceptibility to T- and M-tropic HIV and chemokine-mediated HIV resistance Blood 1999 94 1590 1600 10477684

[B7] Bagnara GP Zauli G Giovannini M Re MC Furlini G La Placa M Early loss of circulating hemopoietic progenitors in HIV-1-infected subjects Exp Hematol 1990 18 426 430 1970962

[B8] Re MC Zauli G Gibellini D Furlini G Ramazzotti E Monari P Ranieri S Capitani S La Placa M Uninfected haematopoietic progenitor (CD34+) cells purified from the bone marrow of AIDS patients are committed to apoptotic cell death in culture AIDS 1993 7 1049 1055 10.1097/00002030-199308000-00004 7691085

[B9] Koka PS Jamieson BD Brooks DG Zack JA Human immunodeficiency virus type 1-induced hematopoietic inhibition is independent of productive infection of progenitor cells in vivo J Virol 1999 73 9089 9097 10.1128/jvi.73.11.9089-9097.1999 PMC112941 10516015

[B10] Neal TF Holland HK Baum CM Villinger F Ansari AA Saral R Wingard JR Fleming WH CD34+ progenitor cells from asymptomatic patients are not a major reservoir for human immunodeficiency virus-1 Blood 1995 86 1749 1756 7544640

[B11] Guilak F Lott KE Awad HA Cao Q Hicok KC Fermor B Gimble JM Clonal analysis of the differentiation potential of human adipose-derived adult stem cells J Cell Physiol 2006 206 229 237 10.1002/jcp.20463 16021633

[B12] Fukuda K Reprogramming of bone marrow mesenchymal stem cells into cardiomyocytes C R Biol 2002 325 1027 1038 10.1016/s1631-0691(02)01524-x 12494500

[B13] Zuk PA Zhu M Ashjian P De Ugarte DA Huang JI Mizuno H Alfonso ZC Fraser JK Benhaim P Hedrick MH Human adipose tissue is a source of multipotent stem cells Mol Biol Cell 2002 13 4279 4295 10.1091/mbc.E02-02-0105 PMC138633 12475952

[B14] Woodbury D Reynolds K Black IB Adult bone marrow stromal stem cells express germline, ectodermal, endodermal, and mesodermal genes prior to neurogenesis J Neurosci Res 2002 69 908 917 10.1002/jnr.10365 12205683

[B15] Toma C Pittenger MF Cahill KS Byrne BJ Kessler PD Human mesenchymal stem cells differentiate to a cardiomyocyte phenotype in the adult murine heart Circulation 2002 105 93 98 10.1161/hc0102.101442 11772882

[B16] Izadpanah R Trygg C Patel B Kriedt C Dufour J Gimble JM Bunnell BA Biologic properties of mesenchymal stem cells derived from bone marrow and adipose tissue J Cell Biochem 2006 99 1285 97 10.1002/jcb.20904 PMC4048742 16795045

[B17] Canque B Marandin A Rosenzwajg M Louache F Vainchenker W Gluckman JC Susceptibility of human bone marrow stromal cells to human immunodeficiency virus (HIV) Virology 1995 208 779 783 10.1006/viro.1995.1211 7747451

[B18] Scadden DT Zeira M Woon A Wang Z Schieve L Ikeuchi K Lim B Groopman JE Human immunodeficiency virus infection of human bone marrow stromal fibroblasts Blood 1990 76 317 322 1695109

[B19] Moses AV Williams S Heneveld ML Strussenberg J Rarick M Loveless M Bagby G Nelson JA Human immunodeficiency virus infection of bone marrow endothelium reduces induction of stromal hematopoietic growth factors Blood 1996 87 919 925 8562963

[B20] Gill V Shattock RJ Scopes J Hayes P Freedman AR Griffin GE Gordon-Smith EC Gibson FM Human immunodeficiency virus infection impairs hemopoiesis in long-term bone marrow cultures: nonreversal by nucleoside analogues J Infect Dis 1997 176 1510 1516 10.1086/514149 9395362

[B21] Sloand EM Young NS Sato T Kumar P Kim S Weichold FF Maciejewski JP Secondary colony formation after long-term bone marrow culture using peripheral blood and bone marrow of HIV-infected patients AIDS 1997 11 1547 1553 10.1097/00002030-199713000-00002 9365758

[B22] Bahner I Kearns K Coutinho S Leonard EH Kohn DB Infection of human marrow stroma by human immunodeficiency virus-1 (HIV-1) is both required and sufficient for HIV-1-induced hematopoietic suppression in vitro: demonstration by gene modification of primary human stroma Blood 1997 90 1787 1798 9292511

[B23] Wang L Mondal D La Russa VF Agrawal KC Suppression of clonogenic potential of human bone marrow mesenchymal stem cells by HIV type 1: putative role of HIV type 1 tat protein and inflammatory cytokines AIDS Res Hum Retroviruses 2002 18 917 931 10.1089/088922202760265597 12230935

[B24] Lawrence DM Durham LC Schwartz L Seth P Maric D Major EO Human immunodeficiency virus type 1 infection of human brain-derived progenitor cells J Virol 2004 78 7319 7328 10.1128/JVI.78.14.7319-7328.2004 PMC434111 15220405

[B25] Freisinger E Cramer C Xia X Murthy SN Slakey DP Chiu E Newsome ER Alt EU Izadpanah R Characterization of hematopoietic potential of mesenchymal stem cells J Cell Physiol 2010 225 888 97 10.1002/jcp.22299 20635396

[B26] Gorry PR Churchill M Crowe SM Cunningham AL Gabuzda D Pathogenesis of macrophage tropic HIV-1 Curr HIV Res 2005 3 53 60 10.2174/1570162052772951 15638723

[B27] Kedzierska K Crowe SM The role of monocytes and macrophages in the pathogenesis of HIV-1 infection Curr Med Chem 2002 9 1893 1903 10.2174/0929867023368935 12369874

[B28] Cassol E Alfano M Biswas P Poli G Monocyte-derived macrophages and myeloid cell lines as targets of HIV-1 replication and persistence J Leukoc Biol 2006 80 1018 1030 10.1189/jlb.0306150 16946020

[B29] Liszewski MK Yu JJ O'Doherty U Detecting HIV-1 integration by repetitive-sampling Alu-gag PCR Methods 2009 47 254 260 10.1016/j.ymeth.2009.01.002 PMC2862469 19195495

[B30] Anderson JS Bandi S Kaufman DS Akkina R Derivation of normal macrophages from human embryonic stem (hES) cells for applications in HIV gene therapy Retrovirology 2006 3 24 10.1186/1742-4690-3-24 PMC1462997 16623949

[B31] Gibellini D Re MC Vitone F Rizzo N Maldini C La Placa M Zauli G Selective up-regulation of functional CXCR4 expression in erythroid cells by HIV-1 Tat protein Clin Exp Immunol 2003 131 428 435 10.1046/j.1365-2249.2003.02095.x PMC1808660 12605695

[B32] Xiao H Neuveut C Tiffany HL Benkirane M Rich EA Murphy PM Jeang KT Selective CXCR4 antagonism by Tat: implications for in vivo expansion of coreceptor use by HIV-1 Proc Natl Acad Sci USA 2000 97 11466 11471 10.1073/pnas.97.21.11466 PMC17223 11027346

[B33] Mondal D Williams CA Ali M Eilers M Agrawal KC The HIV-1 Tat protein selectively enhances CXCR4 and inhibits CCR5 expression in megakaryocytic K562 cells Exp Biol Med (Maywood) 2005 230 631 644 10.1177/153537020523000905 16179731

[B34] Cocchi F DeVico AL Garzino-Demo A Arya SK Gallo RC Lusso P Identification of RANTES, MIP-1 alpha, and MIP-1 beta as the major HIV-suppressive factors produced by CD8+ T cells Science 1995 270 1811 1815 10.1126/science.270.5243.1811 8525373

[B35] Majka M Rozmyslowicz T Lee B Murphy SL Pietrzkowski Z Gaulton GN Silberstein L Ratajczak MZ Bone marrow CD34(+) cells and megakaryoblasts secrete beta-chemokines that block infection of hematopoietic cells by M-tropic R5 HIV J Clin Invest 1999 104 1739 1749 10.1172/JCI7779 PMC409882 10606628

[B36] Li P Burke S Wang J Chen X Ortiz M Lee SC Lu D Campos L Goulding D Ng BL Dougan G Huntly B Gottgens B Jenkins NA Copeland NG Colucci F Liu P Reprogramming of T Cells to Natural Killer-Like Cells upon Bcl11b Deletion Science 2010 329 85 9 10.1126/science.1188063 PMC3628452 20538915

[B37] Marban C Suzanne S Dequiedt F de Walque S Redel L Van Lint C Aunis D Rohr O Recruitment of chromatin-modifying enzymes by CTIP2 promotes HIV-1 transcriptional silencing EMBO J 2007 26 412 423 10.1038/sj.emboj.7601516 PMC1783449 17245431

[B38] Muthumani K Choo AY Premkumar A Hwang DS Thieu KP Desai BM Weiner DB Human immunodeficiency virus type 1 (HIV-1) Vpr-regulated cell death: insights into mechanism Cell Death Differ 2005 12 Suppl 1 962 970 10.1038/sj.cdd.4401583 15832179

[B39] Andersen JL Zimmerman ES DeHart JL Murala S Ardon O Blackett J Chen J Planelles V ATR and GADD45alpha mediate HIV-1 Vpr-induced apoptosis Cell Death Differ 2005 12 326 334 10.1038/sj.cdd.4401565 15650754

[B40] Sedore SC Byers SA Biglione S Price JP Maury WJ Price DH Manipulation of P-TEFb control machinery by HIV: recruitment of P-TEFb from the large form by Tat and binding of HEXIM1 to TAR Nucleic Acids Res 2007 35 4347 4358 10.1093/nar/gkm443 PMC1935001 17576689

[B41] Giraud S Hurlstone A Avril S Coqueret O Implication of BRG1 and cdk9 in the STAT3-mediated activation of the p21waf1 gene Oncogene 2004 23 7391 7398 10.1038/sj.onc.1207972 15286705

[B42] Mansharamani M Graham DR Monie D Lee KK Hildreth JE Siliciano RF Wilson KL Barrier-to-autointegration factor BAF binds p55 Gag and matrix and is a host component of human immunodeficiency virus type 1 virions J Virol 2003 77 13084 13092 10.1128/JVI.77.24.13084-13092.2003 PMC296067 14645565

[B43] Christodoulopoulos I Droniou-Bonzom ME Oldenburg JE Cannon PM Vpu-dependent block to incorporation of GaLV Env into lentiviral vectors Retrovirology 2010 7 4 10.1186/1742-4690-7-4 PMC2831008 20102634

[B44] Serrano-Gomez D Sierra-Filardi E Martinez-Nunez RT Caparros E Delgado R Munoz-Fernandez MA Abad MA Jimenez-Barbero J Leal M Corbi AL Structural requirements for multimerization of the pathogen receptor dendritic cell-specific ICAM3-grabbing non-integrin (CD209) on the cell surface J Biol Chem 2008 283 3889 3903 10.1074/jbc.M706004200 18073208

[B45] Dang Y Davis RW York IA Zheng YH Identification of 81LGxGxxIxW89 and 171EDRW174 domains from human immunodeficiency virus type 1 Vif that regulate APOBEC3G and APOBEC3F neutralizing activity J Virol 2010 84 5741 5750 10.1128/JVI.00079-10 PMC2876610 20335268

[B46] Miller HB Saunders KO Tomaras GD Garcia-Blanco MA Tat-SF1 is not required for Tat transactivation but does regulate the relative levels of unspliced and spliced HIV-1 RNAs PLoS One 2009 4 e5710 10.1371/journal.pone.0005710 PMC2682658 19479034

[B47] Sankale JL Tong Q Hadigan CM Tan G Grinspoon SK Kanki PJ Hotamisligil GS Regulation of adiponectin in adipocytes upon exposure to HIV-1 HIV Med 2006 7 268 274 10.1111/j.1468-1293.2006.00372.x 16630040

[B48] Cotter EJ Chew N Powderly WG Doran PP HIV Type 1 Alters Mesenchymal Stem Cell Differentiation Potential and Cell Phenotype ex Vivo AIDS Res Hum Retroviruses 2010 in press 10.1089/aid.2010.0114 20929345

[B49] Zuk PA Zhu M Mizuno H Huang J Futrell JW Katz AJ Benhaim P Lorenz HP Hedrick MH Multilineage cells from human adipose tissue: implications for cell-based therapies Tissue Eng 2001 7 211 228 10.1089/107632701300062859 11304456

[B50] Izadpanah R Joswig T Tsien F Dufour J Kirijan JC Bunnell BA Characterization of multipotent mesenchymal stem cells from the bone marrow of rhesus macaques Stem Cells Dev 2005 14 440 451 10.1089/scd.2005.14.440 16137233

